# Identifying Variables Influencing Traditional Food Solid-State Fermentation by Statistical Modeling

**DOI:** 10.3390/foods13091317

**Published:** 2024-04-25

**Authors:** Guangyuan Jin, Sjoerd Boeschoten, Jos Hageman, Yang Zhu, René Wijffels, Arjen Rinzema, Yan Xu

**Affiliations:** 1 The Lab of Brewing Microbiology and Applied Enzymology, School of Biotechnology, Jiangnan University, Wuxi 214122, China; g.jin@jiangnan.edu.cn; 2 Bioprocess Engineering, Wageningen University and Research, P.O. Box 16, 6700 AA Wageningen, The Netherlands; sjoerdboeschoten@gmail.com (S.B.); yang.zhu@wur.nl (Y.Z.); rene.wijffels@wur.nl (R.W.); arjen.rinzema@hotmail.com (A.R.); 3 Biometris, Wageningen University and Research, P.O. Box 16, 6700 AA Wageningen, The Netherlands; jos.hageman@wur.nl

**Keywords:** solid-state fermentation, statistical analysis, statistic models, Chinese liquor, ethanol, lactic acid

## Abstract

Solid-state fermentation is widely used in traditional food production, but most of the complex processes involved were designed and are carried out without a scientific basis. Often, mathematical models can be established to describe mass and heat transfer with the assistance of chemical engineering tools. However, due to the complex nature of solid-state fermentation, mathematical models alone cannot explain the many dynamic changes that occur during these processes. For example, it is hard to identify the most important variables influencing product yield and quality fluctuations. Here, using solid-state fermentation of Chinese liquor as a case study, we established statistical models to correlate the final liquor yield with available industrial data, including the starting content of starch, water and acid; starting temperature; and substrate temperature profiles throughout the process. Models based on starting concentrations and temperature profiles gave unsatisfactory yield predictions. Although the most obvious factor is the starting month, ambient temperature is unlikely to be the direct driver of differences. A lactic-acid-inhibition model indicates that lactic acid from lactic acid bacteria is likely the reason for the reduction in yield between April and December. Further integrated study strategies are necessary to confirm the most crucial variables from both microbiological and engineering perspectives. Our findings can facilitate better understanding and improvement of complex solid-state fermentations.

## 1. Introduction

Solid-state fermentation is the cultivation of microbes on moist solid substrates like grains or agro-industrial byproducts and has been used for centuries to produce fermented foods and products, including Chinese liquor [1,2,3,4]. Despite the long tradition of production and the application of a few successfully industrialized processes, especially in the production of enzymes, most solid-state fermentations are still operated in a more traditional and empirical manner compared to submerged fermentation, with less process control [5,6] and consequent fluctuations in product yield and quality. Without knowing the crucial influential variables, control of such a complex process is still infeasible, in particular for a spontaneous process with multi-species involvement and an undefined substrate.

In complex solid-state fermentations, the transport phenomena and transfer coefficients are difficult to measure, with measurement requiring tools typical of chemical engineering. A representative example of such complex solid-state fermentation is the anaerobic fermentation of Chinese liquor (Baijiu in Chinese) [5,7,8]. To improve our understanding of the process, we previously developed a mechanistic model to describe heat and mass transfer in the process [9]. The key assumption in that model is that ethanol production is inhibited only by ethanol and not by (lactic) acid, temperature or lack of starch or water. Therefore, that model predicts that all batch fermentations stop after reaching the same ethanol concentration and thus have the same yield (60% ethanol content after distillation, *v*/*v*). However, industrial data show that the factual yield per batch can fluctuate by a factor of 3.5. Therefore, it seems insufficient to rely solely on such a theoretical model to describe and improve this complex fermentation process.

One approach is to use statistical analysis of process data to identify variables that affect yield. A practical solution is to extract information by statistical modelling of historical process data [10,11,12]. This practice has already been applied in several fermentation systems. For example, multivariate statistical analysis was used for process diagnosis and phase-shift detection during fed-batch fermentation [11,13]. A multivariate monitoring and control approach based on historical process data was demonstrated for monitoring and diagnosis of a small-scale fermentation process [14]. Recently, data-driven dynamic models were built to describe and monitor the dynamic behavior in an industrial batch-fermentation process [15,16].

**Figure 1:**
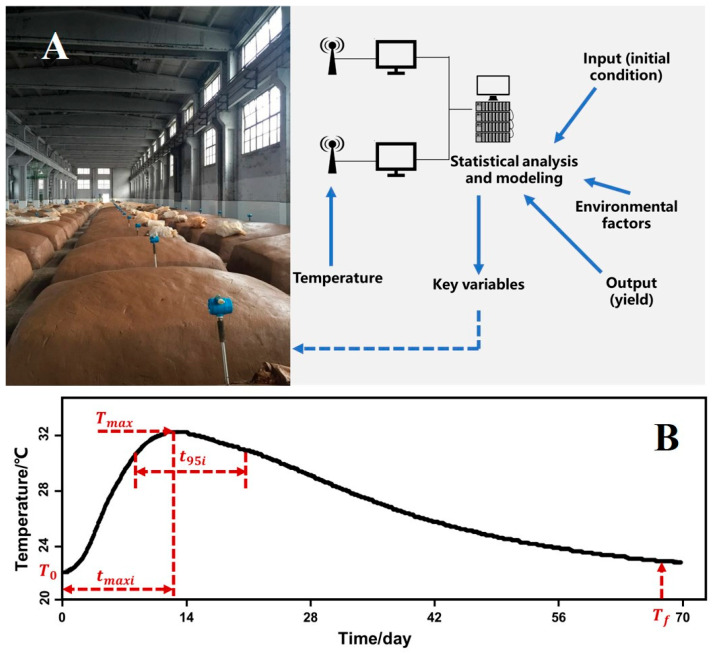
Scheme of temperature sensors and parameters influencing fermentation process (**A**) and the definition of temperature parameters during fermentation (**B**).

Therefore, we here use regression-based modelling with a large dataset obtained from year-round production in a Chinese liquor company with seven factories. We speculate that fluctuations in yield and quality might be caused by any of the operational variables, including temperature, water, substrate and pH, among others. We developed statistical models to correlate the final liquor yield with measured batch inputs and outputs such as starting concentrations of starch, acid and water; temperature during the fermentation; and air temperature outside of the fermentation pit (Figure 1A). This model represents the first use of a large set of data obtained at the factory level and built a statistical model to identify factors influencing a complex solid-state fermentation process. Our findings can not only help to improve understanding of traditional food fermentation, but also improve it the process itself.

## 2. Materials and Methods

### 2.1. Fermentation, Online Measurements and Chemical Analyses

The traditional solid-state fermentation of Chinese liquor was conducted in a Chinese distillery with seven factories, as described elsewhere [5]. In brief, sorghum was first soaked in water at room temperature for 24 h. Soaked sorghum was mixed with fermented substrate from previous batches (1:1, *w*/*w*) and rice husk (1%, *w*/*w*) and then steamed for 2 h. After they had been cooled to room temperature (20 °C), 1350 kg of the steamed materials were mixed with 300 kg Daqu (a type of pre-fermented solid starter mixed with wheat and microbes) [17], then fermented in a pit (an underground and sealed cubical fermentation chamber with a volume around 4 m^3^) for 70 days.

Fermentation temperature was measured hourly by a temperature sensor (MicroDetect Co., Ltd., Tangshan, Hebei Province, China) placed in the vertical and horizontal center of the fermentation pit. The environmental air temperature was measured by the same equipment. Temperature data were collected with computer software based on LabVIEW 2016 (National Instruments Co., Ltd., Austin, TX, USA).

Samples (100 g) were collected before and after fermentation and stored at −20 °C for further analysis. The starting and final concentrations (contents) of starch, water and acids were measured (Table 1). The acid concentration was defined as the quantity of NaOH solution (0.1 M) needed to neutralize 10 g of well-shaken sample-water suspension (*w*/*w*, 1:10) to a final pH of 7.0. The influence of CO_2_ was neglected because the pH of the suspension is around 3 and hardly any CO_2_ can dissolve. Starch content was defined as the weight of starch in 100 g of wet sample and measured through near-infrared spectroscopy (DS2500, Foss, Hillerød, Denmark). Water content was defined as the weight of water in 100 g of wet sample and measured by drying at 60 °C and 133 Pa for 24 h in a vacuum oven (Yiheng Co., Ltd., Shanghai, China).

The final yield was defined as the output of standard fresh liquor (60% ethanol, *v*/*v*) after distillation. The ethanol content was measured using an alcohol meter based on the density and then converted to yield (60% ethanol after distillation, *v*/*v*) through calculation. The weight of fresh liquor was used as an indicator of fermentation output. All the data were gathered and recorded by Microsoft SQL Server 2012 for a whole year’s run (five batches per factory).

### 2.2. Parameter Definitions

Starting and final concentrations were defined as described above (Section 2.1). The starting dates were converted into their corresponding starting month ($M_{s}$). We defined several “feature points” of the temperature dynamics (Figure 1B) to determine whether they could explain the yield and predict fermentation results. Temperature parameters are derived from the temperature data. As shown in Figure 1B and Table 1, starting temperature ($T_{0}$) is defined as the temperature at the first hour, and final temperature ($T_{f}$) is defined as the temperature 48 h before the end of a batch; $T_{max}$ is defined as the maximum temperature in the curve; the time to reach this $T_{max}$ is defined as $t_{maxi}$ in hours. Moreover, to characterize how wide the peak of a curve was, we calculated $t_{95i}$, defined as the time interval in hours around $T_{max}$ when the temperature is higher than 95% of the $T_{max}$. The air temperature ($T_{air}$) was the same in seven factories and was defined as the average temperature over a whole day (24 measurements). We used the average air temperature from 70 days of fermentation ($T_{airavg}$) for regression analysis.

### 2.3. Statistical Analysis

Two main approaches are used for analysis: multiple linear regression models and classifier models. The linear regression models are first-order models, as described in Equation (1), as follows:

(1) $$Y=\beta_{0}+\beta_{1}x_{1}+\beta_{2}x_{2}+\cdots +\beta_{n}x_{n}+\varepsilon$$

where Y is the dependent variable (yield); $x_{1}$, $x_{2}$,…, $x_{n}$ are the variables, and $\beta_{0},$ $\beta_{1}$, $\beta_{2}$, …, $\beta_{n}$ are the regression coefficients; and ε is the residual term. Models were constructed using several combinations of variables (Table 2). Different model specifications were tried and include collinearity checks.

Moreover, 10-fold cross-validation is used to test the predictive capabilities for unknown observations. Hypothesis tests and variance inflation factors were calculated for individual regression coefficients. Regression models were judged on their R^2^, their Q^2^ and the omnibus F-test for the entire model. A higher R^2^ value means that more variances can be explained by the model using training data. Q^2^ values indicate whether a model can predict unseen data well [18]. Q^2^ is calculated using a 10-fold cross-validation. In this test, the data are split into 10 random but nearly equally sized subsets; then. for every partition, a model is fitted. This model is then used to calculate predicted values for the remaining part. These predicted values are used to calculate Q^2^ according to Equation (2), as follows:

(2) $$Q^{2}=1-\left(\frac{Predictive\;residual\;error\;sum\;of\;equares}{Total\;sum\;of\;squares}\right)$$

Good Q^2^ values should be close to their respective R^2^, with a maximum value of 1.

#### 2.3.1. Multiple Regression

The omnibus F-test for the regression model indicates whether a model has any statistical significance. Individual hypothesis tests and their resulting *p*-values for regression coefficients indicate whether a coefficient has a significant positive or negative relationship in the model. A significance level of *p*-value of 0.05 is used throughout this study. Variance inflation factors are used to check for collinearity. These values are calculated with Equation (3), as follows:

(3) $${VIF}_{i}=\frac{1}{1-R_{i}^{2}}$$

Here, ${VIF}_{i}$ indicates the variance inflation factors value for the ith variable. $R_{i}^{2}$ is the variance explained when variable i is predicted using all other variables. Collinearity occurs when two or more variables are closely correlated with each other. This decreases the interpretability of their coefficient estimates, as one could be expressed as the other. Moreover, significant coefficients can be missed as well because otherwise significant *p*-values can be greater than 0.05 due to collinearity. Therefore, it is important to address this issue by, for example, dropping some of these parameters. A variance inflation factor value greater than 5 indicates a problematic amount of collinearity [19].

#### 2.3.2. Classifier Model

The classifier model is a decision-tree model used to characterize different yield classes and to simplify representation of the data [20]. This is accomplished by manually assigning a class label to certain ranges of yield. The decision tree tries to find a set of rules based on selected parameters to split the data into their corresponding classes. Every rule is a true or false condition. For example, if a parameter value is over a certain threshold, then that observation is assigned to node 2; it is otherwise assigned to node 3. This process repeats for every node until the algorithm ends. Nodes show what proportion of the original data they contain. The separation and percentage of original data among the nodes indicate how effective a rule is.

## 3. Results and Discussion

### 3.1. Data Distribution

**Figure 2:**
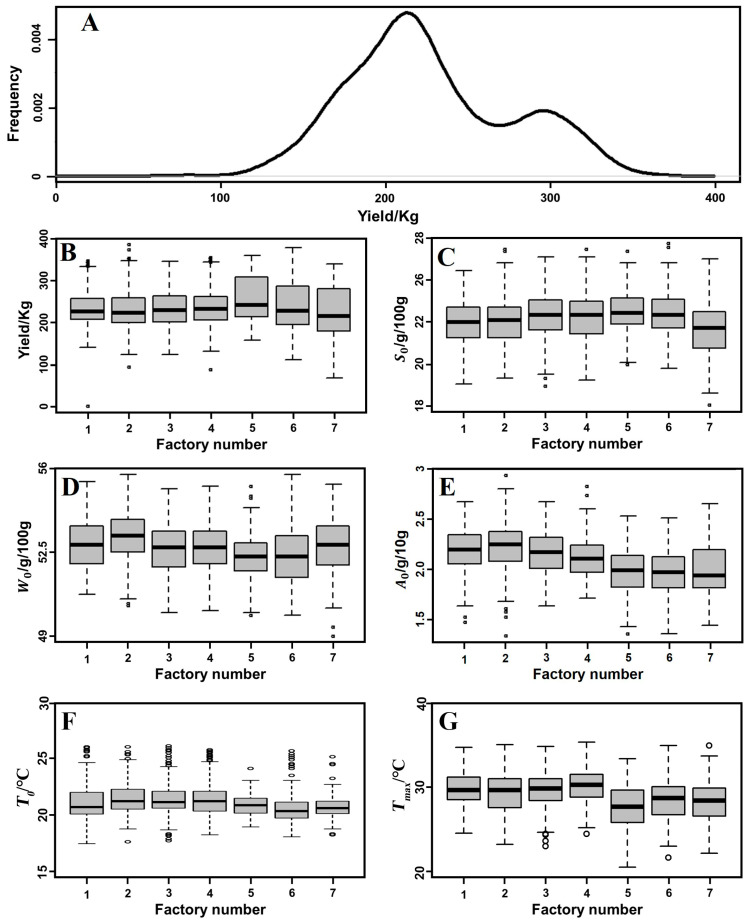
Frequency plots of (**A**): yield production per batch for all seven factories (*N* = 1622); also shown are the distributions of different parameters per factory, including (**B**) yield, (**C**) initial values of starch, (**D**) water and (**E**) acid content, (**F**) initial temperature and (**G**) maximum temperature in the pit, shown separately.

**Analysis of data distribution in the real fermentation process.** After outliers and incomplete entries were removed, the data set contained 1622 batch runs. As shown in Figure 2A, the frequency distribution of the yield is bimodal instead of normal. The low-yield peak contains more data than the high-yield peak; the distance between the peaks is around 280 kg of liquor per batch.

Figure 2B–G also shows box plots representing the distributions of the final yields, initial starting concentrations, initial temperatures and peak temperatures for each factory. The medians and interquartile ranges of the factories align rather well for all parameters except for the yield. The medians of the yields are almost the same for all factories, but the values of the 75th percentile (top of the boxes) differ: factories 5, 6 and 7 have a skewed yield distribution, with more high-yield batches than factories 1 through 4 have. All factories have a bimodal distribution. This result suggests that all factories operate similarly and have similar yield ranges.

Note that starch and water content do not sum to a mass balance of 100% because of the partial recycling of fermented material (see Materials and Methods).

### 3.2. Descriptive Statistics

**Figure 3:**
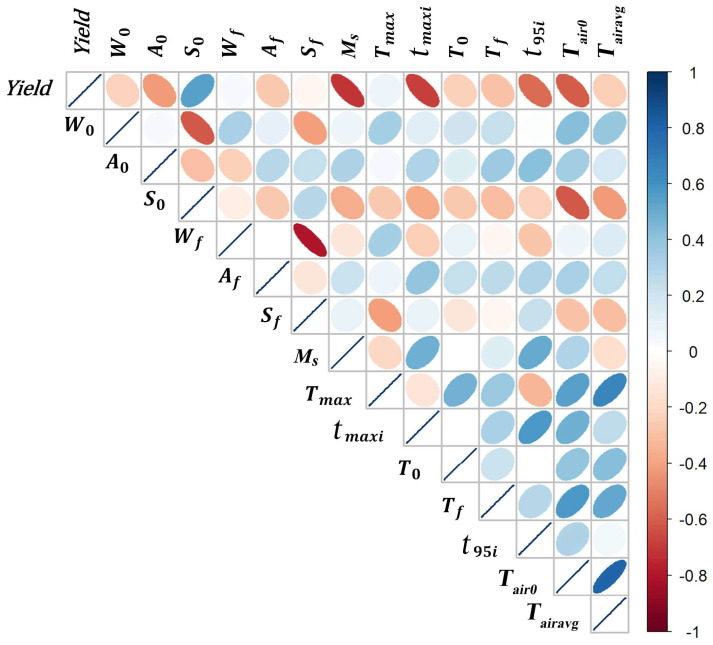
Correlation plot of variables used in analysis. Blue color indicates a positive correlation (correlation coefficient from 0 to 1) whereas red color shows a negative correlation (correlation coefficient from −1 to 0).

**Overview of general associations between variables**. Figure 3 shows the correlation between the liquor yield and single variables. The thinner the ellipsoid, the stronger the correlation. The yield is correlated (R > 0.5) with the initial starch content ($S_{0}$), the starting month ($M_{s}$), the time needed to reach the maximum pit temperature ($t_{maxi}$), the duration of the high-temperature period ($t_{95i}$) and the initial air temperature ($T_{air0}$), but has no meaningful correlation with any other single variable.

The positive correlation of the yield with the initial starch content seems logical because the inclusion of more starch can result in more ethanol. However, a large amount of starch was still available at the end of all fermentations (the average final starch content was around 12 g/100 g wet substrate). Considering that starch is thus unlikely to be a limiting factor, other factors must have stopped the ethanol production.

The strongest correlation is that between the yield and the month when a batch fermentation started. This finding agrees with the industrial experience that the yield (as defined earlier) is always higher in the winter (December to February). In practice, some factories even stop producing during the summer (June to August). Several factors associated with the starting month could explain this pattern: microbial diversity, air temperature, raw material, etc.

**Figure 4:**
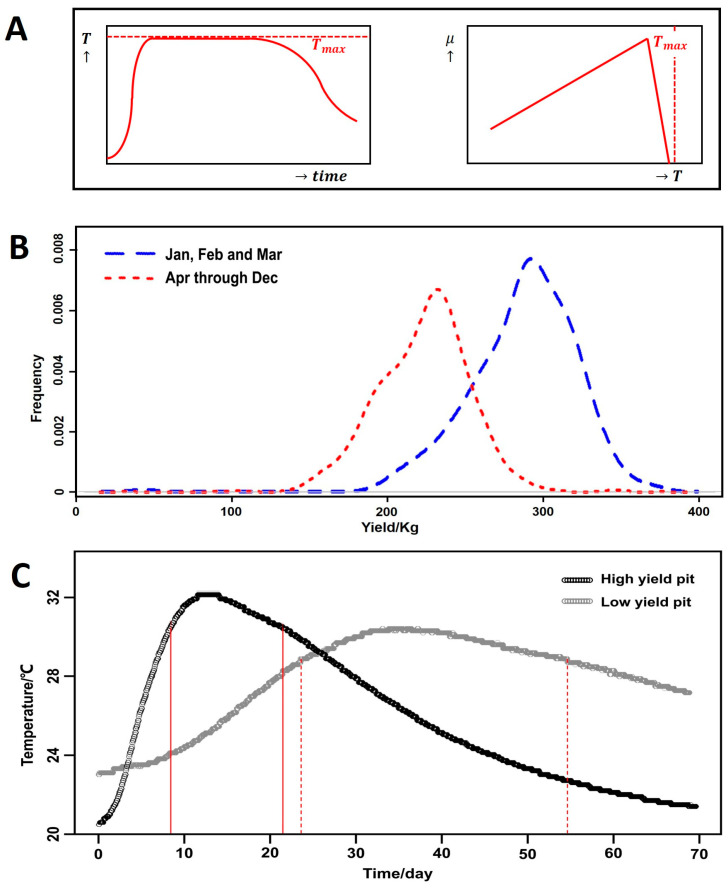
(**A**) Schematic temperature profile for solid-state fermentation with insufficient cooling (**left**), and temperature dependence of the specific growth rate (**right**). (**B**) Classifier model result for typical high-yield (>280 kg) and low-yield (<280 kg) pits. The red lines indicate the time interval during which the pit temperature is at least 95% of the maximum temperature in the pit (*t*_95*i*_). Frequency plot of yield in January through March (*N* = 420) and in the rest of the year (*N* = 1202). (**C**) Frequencies are based on the number of batches in the group.

A striking result is that the yield is not correlated with the maximum substrate temperature ($T_{max}$). This finding agrees with the absence of any signs of overheating in the observed temperature profiles (Figure 4A). After a rapid initial increase of the temperature, the bioreactor reaches a phase in which the temperature is (almost) constant because it is close to the maximum temperature for microbial growth (Figure 4A). Later, the temperature decreases because other factors such as a substrate component or an inhibitory microbial product might be limiting microbial growth. Therefore, the correlation of the yield with the time needed to reach the peak temperature ($t_{maxi}$) and the length of the high-temperature period ($t_{95i}$) may be inversely causally related: heat production depends mainly on ethanol production [9]. Consequently, any factor that slows down ethanol production will prevent the fermentation pit from heating.

The yield is negatively correlated with initial and final acid contents, but the correlation is not as strong as expected. It is known that acids can inhibit yeast growth [21] and the industry regards higher acidity as one of the primary causes of process failure. However, the initial and final acid contents show little variation—although they do differ from one another—and this may have obscured the effect of acid content. In addition, it is possible that there is a non-linear effect or that the effect of the final acid content is related to the time at which it is reached.

A higher initial air temperature is associated with a lower yield, but the yield is not significantly correlated with the average air temperature during the fermentation. The effect of the initial air temperature is difficult to explain because heat exchange between the air and the substrate is poor [9]. Cooling depends mainly on the temperature of the soil surrounding the pit, which follows the air temperature with a significant delay [22]. Therefore, the effect of the initial air temperature is probably indirect. This value could be a proxy for the starting month because the best months for fermentation have the lowest air temperatures.

Despite some non-significant (e.g., $T_{air0}$ and $S_{0}$) and weak correlations (e.g., $A_{0}$ and $W_{0}$), some of the correlations between different variables are notable and logical. For example, the starch and water contents are linked strongly because starch and water are the two main components of the wet substrate. The average air temperature ($T_{airavg}$) is not correlated with the starting month because the regression is based on linear correlation, while the relationship of average air temperature to starting month is not linear.

### 3.3. Correlation of Yield with All Variables except Final Contents

If we take into account all variables except the final contents of water, starch and acid ($W_{f}$, $S_{f}$ and $A_{f}$), the correlation can explain 82% of the yield variance (Table 2). This model has a Q^2^ of 0.81, a value close to the R^2^ value, indicating that the model is reliable. We did not include these final contents because of their weak correlation with the yield (Figure 3) and because they currently cannot be adjusted during fermentation in industrial practice. Note that the absolute values of coefficients do not indicate the importance of the corresponding variables because the variables have very different numerical values, although the sign of the coefficients indicates whether a variable has a positive or negative effect on the final yield (in the presence of the other variables in the model).

For most variables, the direction of their effect on the yield is the same as shown in Figure 3. However, there are two exceptions:

- The initial acid content ( $A_{0}$) has a very significant negative effect (*p* < 0.001) in combination with other variables.
- The maximum substrate temperature has a significant positive coefficient. This finding indicates that no overheating occurred. As discussed earlier, this result does not indicate that heating stimulates ethanol production.

### 3.4. Model Based on Initial Conditions

**Model based on initial acids, starch and water content.** The starting contents of acids, starch and water ($A_{0}$, $S_{0}$ and $W_{0}$) and the initial temperature ($T_{0}$) are always adjusted in the industrial standard before a fermentation batch is started [23]. This practice likely explains why a yield model using the starting contents could explain only a small proportion of the variance in yield (R^2^ = 0.36) and why introducing initial temperature ($T_{0}$) resulted in only a slight improvement to R^2^, raising its value to 0.37 (Table 2). All coefficients of the model are statistically significant (*p* ≤ 0.1), and this model is easy to use in practice, as the values of all parameters can be adjusted at the start of a batch. At the same time, parameters other than these initial ones also affect the variation in yield in practice.

Table 2 also shows collinearity variance inflation factor values that are all below the threshold of 5, meaning that no important collinearity is present even though there are some correlations between parameter pairs like $S_{0}$ and $A_{0}$ or $S_{0}$ and $W_{0}$ (Figure 3).

### 3.5. Model Based on Temperature Variables

**Model based on the defined temperature features.** The temperature curve is often used as an indicator of fermentation performance in the liquor-production industry. Experience shows that temperature curves of high-yield and low-yield batches differ markedly. The result of the regression analysis in Table 2 shows that pit-temperature-related variables can predict 56% of the yield variance. The maximum substrate temperature ($T_{max}$) and the final substrate temperature ($T_{f}$) have small coefficients with large error margins and low significance. The initial substrate temperature ($T_{0}$) has a coefficient 10 times larger, with a small error margin and a high significance. This result indicates that $T_{0}$ is the only important temperature of these three. The negative coefficient of $T_{0}$ may be due to a correlation with the starting month. The positive coefficient of $T_{max}$ again indicates that overheating is unlikely to have been a problem in the studied batch runs.

The coefficients of $t_{maxi}$ and $t_{95i}$ are much smaller than those of the other temperature variables because these time indicators are expressed in hours, so the numbers are much greater than those representing temperatures. They have a very significant effect (*p* < 0.001). Batches with higher yield tend to reach the maximum temperature sooner and remain above 95% of their maximum temperature for a shorter time. As mentioned before, a rapid temperature increase could be the result of rapid microbial growth and fermentation, instead of the other way around. Previously, we found that the temperature peak occurred when the ethanol production stopped due to product inhibition [9]. Therefore, if the temperature peaks and the yield vary, there must be other limiting factors involved.

The classifier model suggests a yield boundary of 280 kg per batch. Batches with a yield below 280 kg were classified as low-yield, and those with a yield above 280 kg were classified as high-yield. The decision-tree model confirms visual differences between typical temperature curves for both high-yield and low-yield fermentations (Figure 4B). This finding agrees well with industrial experience. For example, temperature can indicate fermentation performance, including final yield. A slow temperature increase and a smaller temperature difference between $T_{max}$ and $T_{0}$ correspond logically to slow ethanol production. Furthermore, the decision-tree model confirms that a quick transition to the maximum temperature is important; as shown in Figure 4B, a large proportion of high-yield batches reach their peak temperature early. This finding indicates that the temperature curve ($T_{0}$, $t_{maxi}$, $T_{max}$, $t_{95i}$ and $T_{f}$) can be an indicator of fermentation performance, even though the R^2^ value of the regression model is only 0.56. As discussed above, temperatures are unlikely to be the cause of low yield.

### 3.6. Model Based on Starting Month

**Model based on the manufacturing starting month.** Using the starting month of the batches as the sole independent variable, the model has an R^2^ value of 0.72 and a Q^2^ value of 0.72 (Table 2). Therefore, the starting month is by far the most important variable. To check the effect of starting month, the data set was separated into two groups based on the classifier model: one group consisting of batches started in January, February and March, and the other consisting of batches started in all other months. Figure 4C shows the yield frequency distributions of these two groups. Splitting the data based on high-yield months versus low-yield months results in two near-normal distributions. This result indicates that some (yet) unknown environmental or seasonal factors may play an important role.

The decision-tree model confirms that the starting month is an important factor for distinguishing between high-yield and low-yield batches. All batches are assigned a class based on their yields. In the decision-tree model, one simple rule in the top node splits most of the data. This rule is based on the starting month: a starting month of January, February, or March indicates that the batch is probably high-yield, as 82% of the batches in this node are high-yield. However, this node still contains 18% low-yield batches, which must be the result of the overlap of the unimodal distributions. Therefore, some variation can still be found within these high-yield months.

### 3.7. Explaining the Seasonal Effect

**Figure 5:**
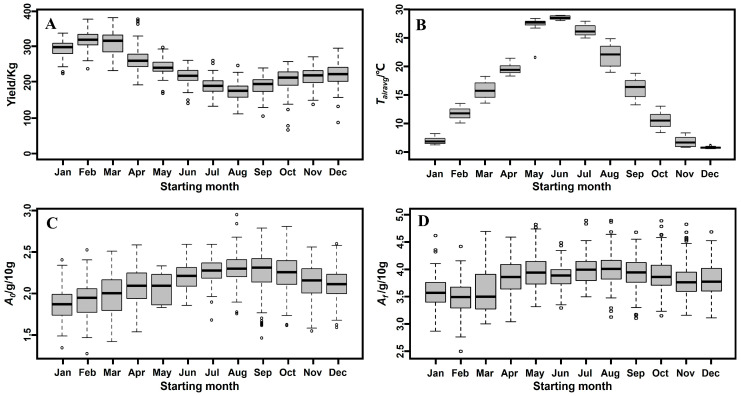
Box plots for the distribution of (**A**) yield, (**B**) average air temperature, (**C**) initial acid content and (**D**) final acid content by starting month.

**Identify the driver of the seasonal change in output. **Figure 5 shows the variations in yield, average air temperature, initial acid content and initial starch content over the year. There are strong correlations between the monthly medians of the yield and the medians of average air temperature, initial acid content and initial starch content (Figure 5). However, the correlation with all values is poor (Figure 3). This result is to be expected because higher and lower values cancel each other if we consider the median values only.

Air temperature is the most obvious variable that changes with the season (Figure 5B). The model with the initial and average air temperatures together ($T_{air0}$ and $T_{airavg}$) has an R^2^ of 0.55 (Table 2), but the model with average air temperature alone has an R^2^ of 0.05. Only the initial air temperature has an effect. The air temperature is associated with the cooling of the pits, either directly or indirectly via the soil temperature [22], affecting cooling [9]. However, there are no signs that overheating occurred during the fermentation of any of the batches. Therefore, air temperature cannot explain the seasonal yield variation.

Lactic acid is one of the main products of Chinese liquor fermentation and can reach concentrations as high as 2.0 g/100 g wet substrate [24,25]. A lactic acid content between 0.2 and 0.8% (*w*/*v*) induces stress in yeasts [26]. If lactic acid bacteria are dominant at the beginning of a batch process, they can inhibit the whole fermentation process [27,28]. Moreover, lactic acid bacteria form a greater percentage of the total microbial biomass in the fermentation pit during the summer compared to the winter [29,30]. As shown in Figure 5, both the initial and final acid content values are lower in January, February and March compared to other months. This result indicates that lactic acid could be a significant factor, along with starting month.

Previously, we built a simple model to describe the traditional solid-state fermentation process, and we assumed that lactic acid bacteria start producing lactic acid after ethanol has reached its maximum concentration [9]. If lactic acid production starts earlier or if the initial content is higher, lactic acid might affect the yeast. To understand how this interaction could affect the yield, we introduced lactic acid inhibition of yeast to our published model [9], as shown in Equation (4):

(4) $$\mu_{E}=\mu_{EMAX}\left(1-\frac{c_{E}}{c_{EMAX}}\right)\left(1-\frac{c_{L}}{c_{LMAX}}\right)$$

where $c_{E}$ is the ethanol concentration (Cmol/m^3^ wet substrate), $c_{L}$ is the lactic acid concentration (Cmol/m^3^ wet substrate), $c_{EMAX}$ is the maximum ethanol concentration (Cmol/m^3^ wet substrate), $c_{LMAX}$ is the maximum lactic acid concentration (Cmol/m^3^ wet substrate), $\mu_{E}$ is the specific growth rate of yeast and $\mu_{EMAX}$ is the maximum specific growth rate.

**Figure 6:**
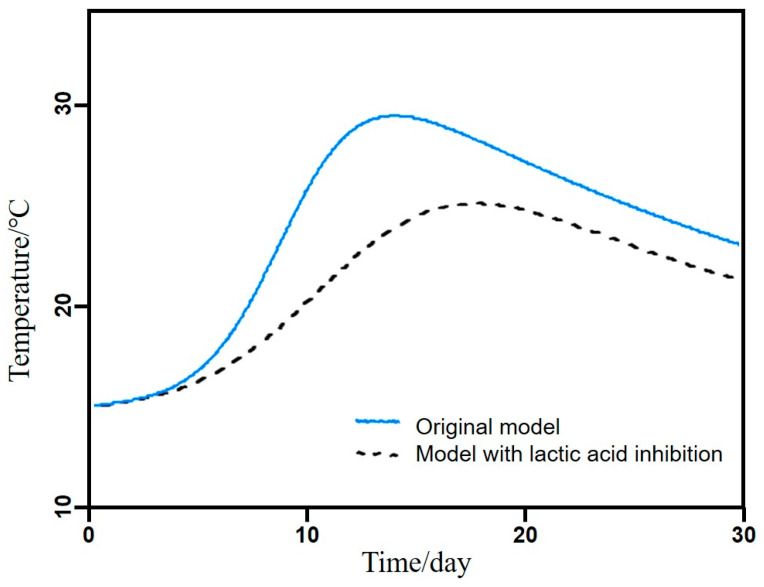
Original temperature model and temperature model with lactic acid inhibition.

Using the previously found $c_{EMAX}$ value and applying the $c_{LMAX}$ value found for lactic acid bacteria to yeast, we simulated the effect of a higher initial content of lactic acid and/or lactic acid bacteria. In both cases, lactic acid inhibited yeast before ethanol reached its maximum concentration. The simulated temperature curves (Figure 6) are similar to those shown in Figure 4C. This result shows that a higher initial content of lactic acid or lactic acid bacteria can explain the differences in yield between warm and cold months. If these higher initial contents are found during spring, summer and autumn, this phenomenon could explain the observed effect of the starting month.

In our previously published model, we also assumed that starch hydrolysis is not rate-limiting [9]. We could also obtain simulated temperature profiles like those shown in Figure 6 by adding a low rate of starch hydrolysis to the model. This effect could be caused by seasonal variation in the quality of the Daqu starter used as the starter culture. However, we do not yet have data to support the hypotheses that low liquor yield is caused by lactic acid or by slow starch hydrolysis. Integrated study strategies combining both microbiological and engineering insights are needed to confirm the exact mechanisms that explain the crucial influence of the starting month.

## 4. Conclusions

To identify the variables influencing a complex solid-state fermentation process, we built statistical models based on available industrial data, multilinear regression and classifier models. Model based on starting concentrations and temperature profiles cannot produce reliable predictions. The starting month (warm versus cold months) is the most important variable, but not because of the ambient temperature. The mechanism could involve the inhibition of yeast by lactic acid bacteria between April and December. Future studies should use integrated strategies combining both microbiological and engineering insights to identify the exact mechanisms that explain the crucial influence of the starting month.

## Figures and Tables

**Table 1:** Parameter description.

Parameter	Description	Unit
*A*_0_	Starting concentration of acid	g/10 g wet substrate
*A_f_*	Final concentration of acid	g/10 g wet substrate
*S*_0_	Starting concentration of starch	g/100 g wet substrate
*S_f_*	Final concentration of starch	g/100 g wet substrate
*W*_0_	Starting content of water	g/100 g wet substrate
*W_f_*	Final content of water	g/100 g wet substrate
*s_mn_*	Starting month	NA
*s_wk_*	Starting week	NA
*T*_0_	Starting temperature	°C
*T_f_*	Final temperature	°C
*T_max_*	Maximum temperature	°C
*t_maxi_*	Time to maximum temperature	h
*t*_95*i*_	Time at 95% of the maximum temperature	h
*a_t_*	Total area under temperature curve	NA
*a_p_*	Area under temperature curve before maximum temperature is reached	NA
*T_soil_*	Soil temperature	°C

NA: not available.

**Table 2:** Summary of model results.

Parameters	Coefficient ± Standard Error						
*β*_0_	114.2 ± 5.0 ***	−540.7 ± 92.8 ***	526.5 ± 13.5 ***	−56.3 ± 13.0 *	290.9 ± 2.3 ***	273.2 ± 3.8 ***	−46.6 ± 6.2 *
*W*_0_	1.6 ± 0.3	8.2 ± 1.3 *	0	1.4 ± 1.1	0	0	2.9 ± 1.0
*A*_0_	−9.5 ± 0.6 ***	−42.5 ± 2.3 ***	0	−10.0 ± 0.9 ***	0	0	−8.5 ± 0.7 ***
*S*_0_	4.3 ± 0.3 ***	23.2 ± 1.2 ***	0	14 ± 0.9 ***	0	0	14 ± 0.9 ***
*T*_0_	−5.0 ± 0.5 *	−3.7 ± 0.8 ***	−11.0 ± 0.6 ***	−7.0 ± 0.3 ***	0	0	−7.0 ± 0.5 ***
*t_maxi_*	−0.1 ± 0.0 ***	0	−0.2 ± 0.0 ***	−0.1 ± 0.0 ***	0	0	−0.1 ± 0.0 ***
*T_max_*	4.1 ± 0.5 *	0	1.2 ± 0.3 *	2.5 ± 0.6 *	0	0	2.1 ± 0.6 *
*t*_95*i*_	−0.1 ± 0.0 ***	0	−0.1 ± 0.0 ***	−0.1 ± 0.0 ***	0	0	−0.1 ± 0.0 *
*T_f_*	−0.5 ± 0.11	0	0.6 ± 0.3	0.48 ± 0.2	0	0	0.2 ± 0.1
*M_s_*	−37.3 ± 2.0 ***	0	0	0	−66.0 ± 1.9 ***	0	0
*T_air_*_0_	−1.3 ± 0.1 *	0	0	0	0	−7.8 ± 0.4 **	−1.6 ± 0.1 *
*T_airavg_*	−0.3 ± 0.1	0	0	0	0	−1.6 ± 0.2	0.4 ± 0.2
R^2^	0.82	0.37	0.56	0.63	0.72	0.55	0.71
Q^2^	0.81	0.37	0.56	0.63	0.72	0.55	0.71
VIF	3.05	1.16	1.46	1.66	2.08	1.00	2.01

Significance codes: *: *p* < 0.05, **: *p* < 0.01, ***: *p* < 0.001. A coefficient of “0” indicates that the parameter was not taken into account for modeling.

## Data Availability

The original contributions presented in the study are included in the article, further inquiries can be directed to the corresponding author.
